# Supplementary material for: Scalable photonic sources using two-dimensional lead halide perovskite superlattices
Source: Nat Commun. 2020 Jan 20;11:387. doi: 10.1038/s41467-019-14084-3 (PMC6971243; doi:10.1038/s41467-019-14084-3)
Supplement: Supplementary file 1 — Supplementary Information [file 41467_2019_14084_MOESM1_ESM.pdf]

Scalable Photonic Sources Using Two-Dimensional Lead Halide Perovskite Superlattices

Jakub Jagielski[1], Simon F. Solari[1], Lucie Jordan[1], Declan Scullion[2], Balthasar Blülle[3], Yen-Ting Li[4,5], Frank Krumeich[6], Yu-Cheng Chiu[4,7], Beat Ruhstaller[3,8], Elton J. G. Santos[2], and Chih-Jen Shih[1]*

[1] *Institute for Chemical and Bioengineering, ETH Zürich, 8093 Zürich, Switzerland.*

[2] *School of Mathematics and Physics, Queen's University Belfast, BT7 1NN, United Kingdom.*

[3] *Fluxim AG, 8400 Winterthur, Switzerland.*

[4] *Department of Chemical Engineering, National Taiwan University of Science and Technology, Taipei 10607, Taiwan.*

[5] *National Synchrotron Radiation Research Center, Hsinchu 30076, Taiwan.*

[6] *Laboratory of Inorganic Chemistry, ETH Zürich, CH-8093 Zürich, Switzerland.*

[7] *Advanced Research Center for Green Materials Science and Technology, National Taiwan University, Taipei 10617, Taiwan.*

[8] *Institute of Computational Physics, Zurich University of Applied Sciences (ZHAW), 8400 Winterthur, Switzerland.*

* Author to whom all correspondence should be addressed. Email: chih-jen.shih@chem.ethz.ch

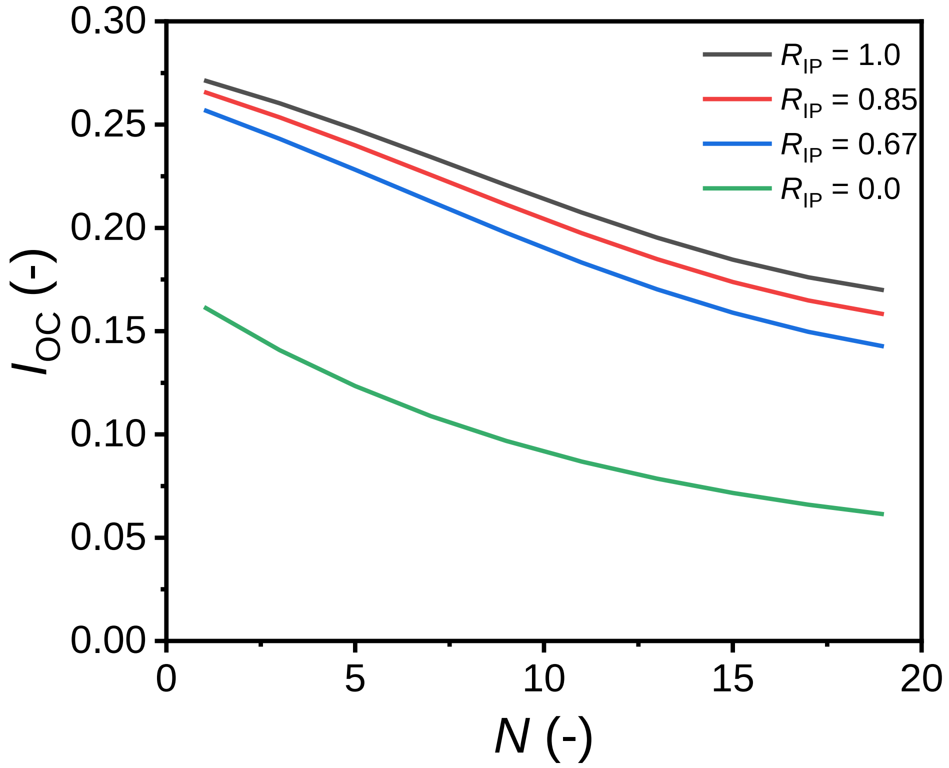

Supplementary Figure 1 | Outcoupling efficiency as a function of superlattice thickness *N* and dipole orientation. The calculated percentage of energy dissipation for $k/k_0 \leq 1$, I_{OC}, in air/superlattice/substrate dielectric stacks as a function of N. Due to the cavity effect and increased fraction of radiation reflected from glass, I_{OC} decreases with N for the N range considered here. Also as expected, an increase of R_{IP} increases I_{OC}, highlighting the emission directionality.

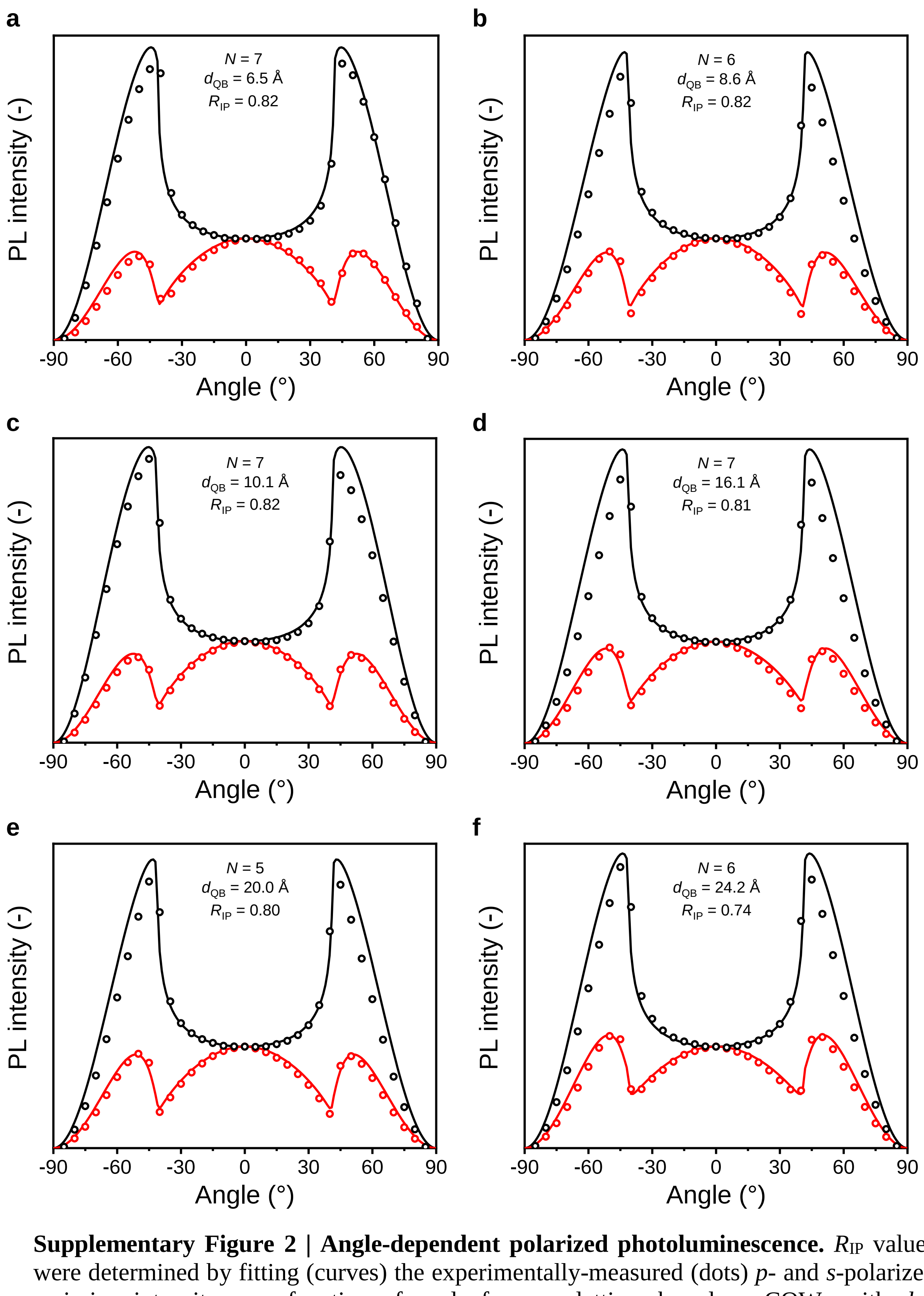

Supplementary Figure 2 | Angle-dependent polarized photoluminescence. R_{IP} values were determined by fitting (curves) the experimentally-measured (dots) *p*- and *s*-polarized emission intensity as a function of angle for superlattices based on CQWs with d_{QB} increasing from **a** to **f**. It is observed that d_{QB} does not change the fitted R_{IP} values, with the exception of superlattices of d_{QB} = 24.2 Å, presumably due to a smaller lateral size (see Supplementary Fig. 11).

a

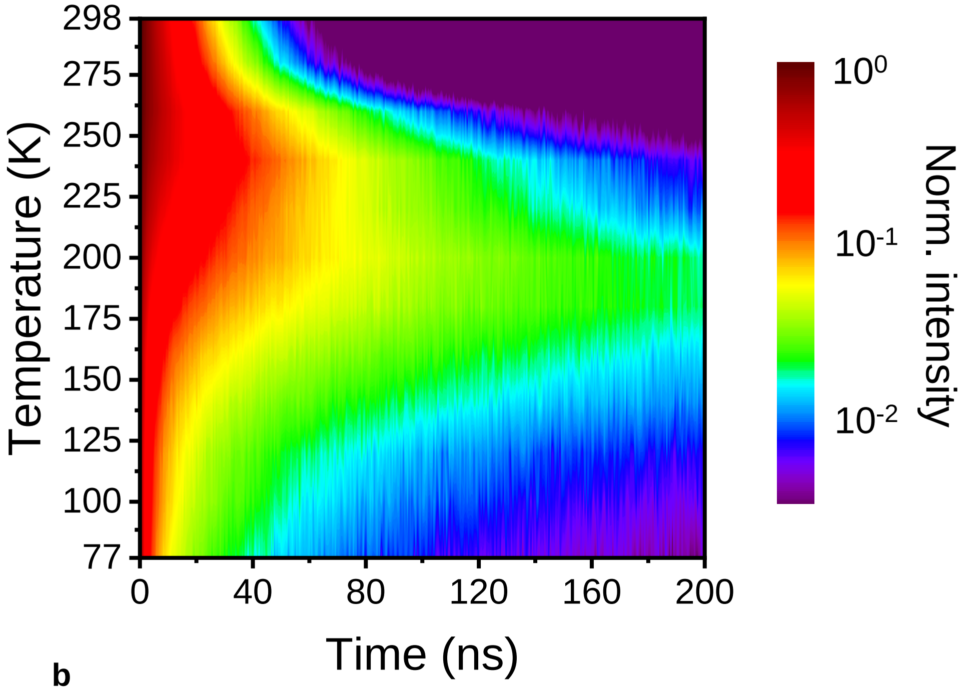

b

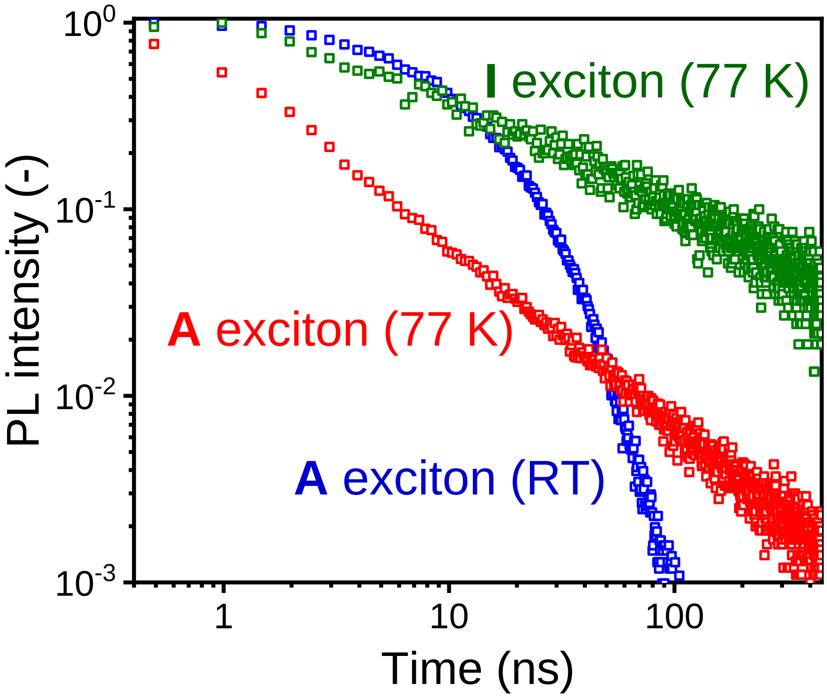

Supplementary Figure 3 | Temperature-dependent TRPL analysis. a, Time-resolved PL intensities as a function of temperature for a superlattice of CQWs with d_{QB} = 25.6 Å. The initial monoexponential decay at room temperature gradually extends upon cooling down. A maximum lifetime is observed between 180 and 220 K. However, after this temperature threshold, the radiative recombination becomes significantly faster, especially at the prompt stage. **b**, The same data set as in Fig. 3b is shown in log-log scale. At cryogenic conditions the **A** exciton emission decay clearly follows the power law (red) with a proportion $I_{PL}(t) \sim t^{-1.0}$.

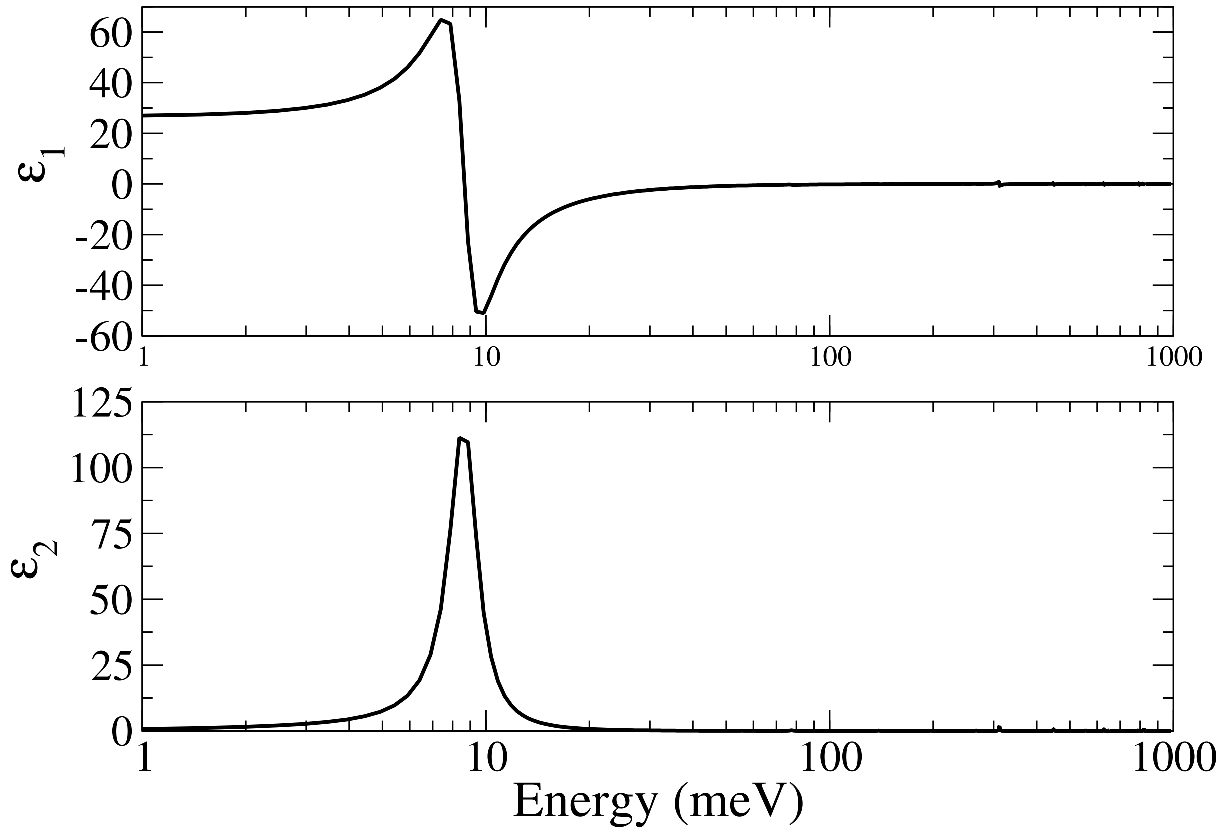

Supplementary Figure 4 | Dielectric response. DFT calculated real (ε_1) and imaginary (ε_2) parts of the dielectric function of bulk $MAPbBr_3$ with ionic contributions included.

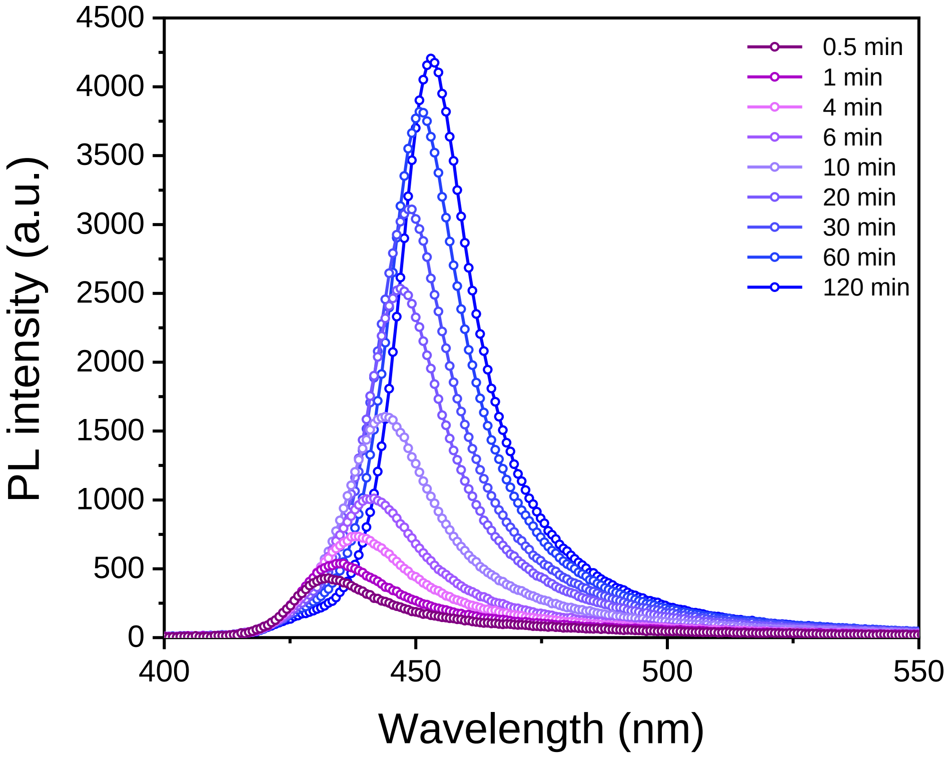

Supplementary Figure 5 | Formation of $MAPbBr_3$ *n* = 3 CQWs. Photoluminescence spectra measured for a series of aliquots, which were withdrawn from the reaction mixture at different times without any purification. Directly after all reagents are mixed, a formation of thinner *n* = 1 CQWs is observed, which is evidenced by the position of the emission maximum at ~435 nm. It is then followed by a gradual growth of *n* = 3 CQWs, which results in a spectral red-shift towards ~455 nm as well as an intensity increase due to enhanced η_{PL}.

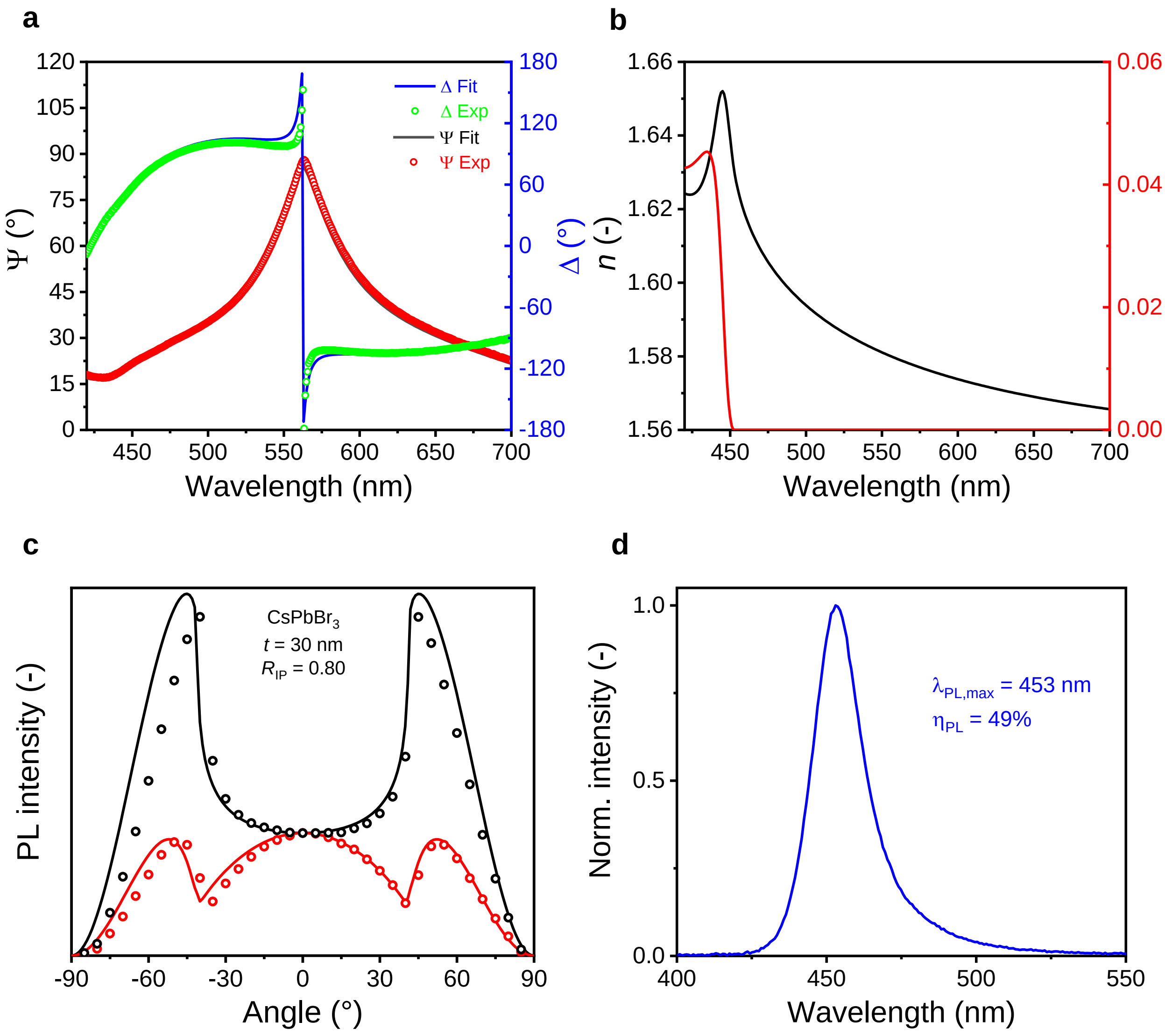

Supplementary Figure 6 | Analysis of a superlattice film based on $CsPbBr_3$ *n* = 3 CQWs. An analogous superlattice composed of *n* = 3 $CsPbBr_3$ CQWs was fabricated and analyzed by ellipsometry (**a** and **b**) and angular PL measurements (**c**). With R_{IP} of 0.8, it behaves nearly identical to the $CH_3NH_3PbBr_3$ counterparts, suggesting that the interlayer screening is not influenced by the molecular dipole dielectric response ε_{dip}. As we discussed in the main text, the interlayer screening is mainly contributed by both ε_{optic} and ε_{ion} at room temperature. Steady-state PL characteristics are also similar to those obtained for hybrid CQWs (**d**)

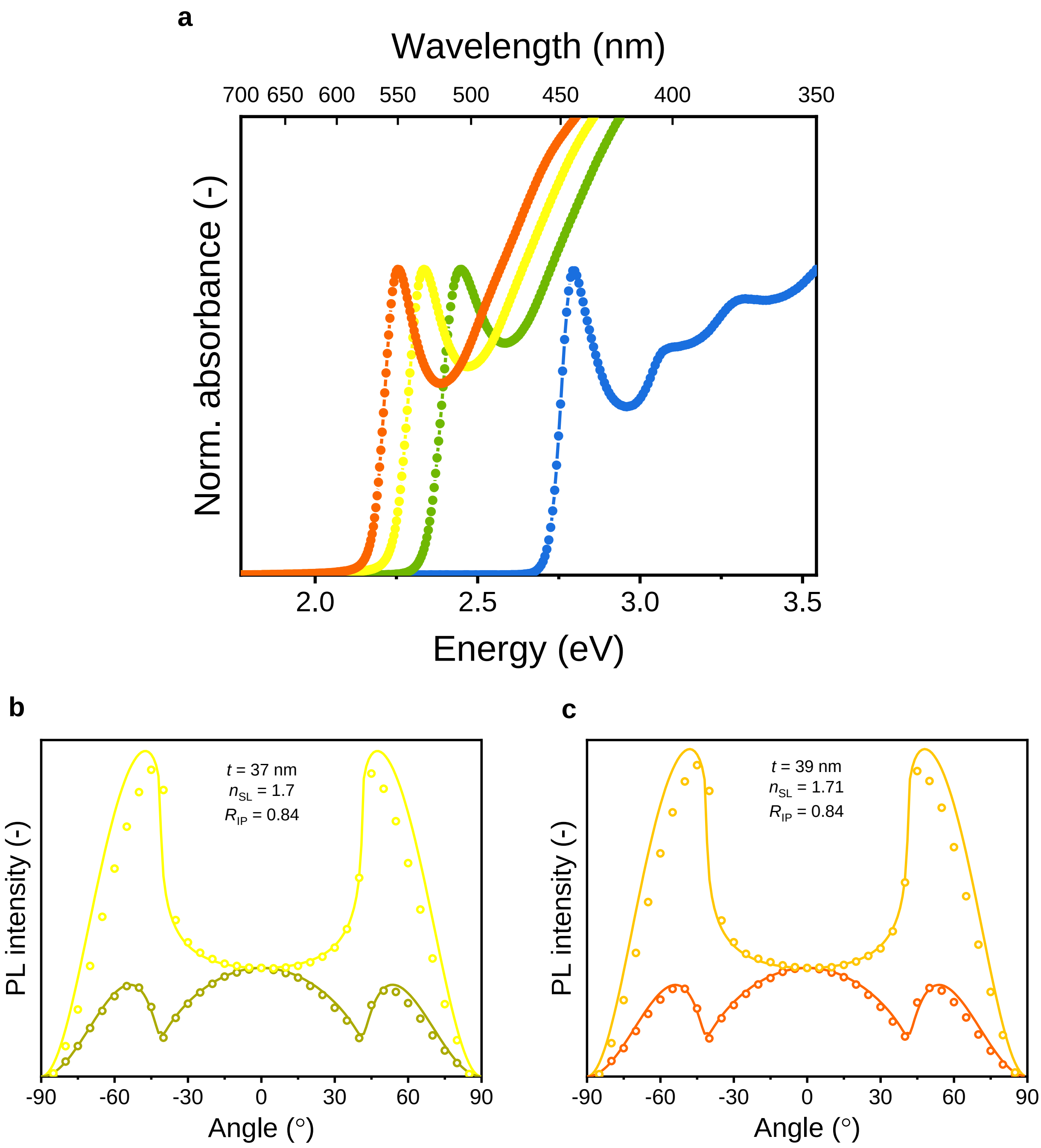

Supplementary Figure 7 | Spectroscopic analysis for CQW superlattices after AE. a, Normalized optical absorption spectra indicate the change in band gap energy upon gradual introduction of iodide ions into the perovskite crystal lattice. The excitonic feature near band gap energy presents in all colloidal dispersions, which is a direct evidence of quantum confinement effect. Angle-dependent PL spectra recorded for the superlattices of CQWs with d_{QB} = 25.6 Å after AE, which are emitting in yellow (**b**) and orange (**c**) spectral region. Fitting of the experimental data yields R_{IP} parameter of 0.84 in both cases. The film thicknesses correspond to stacking numbers N = 7. Here, a constant value of n_{SL} from a non-absorbing region was applied.

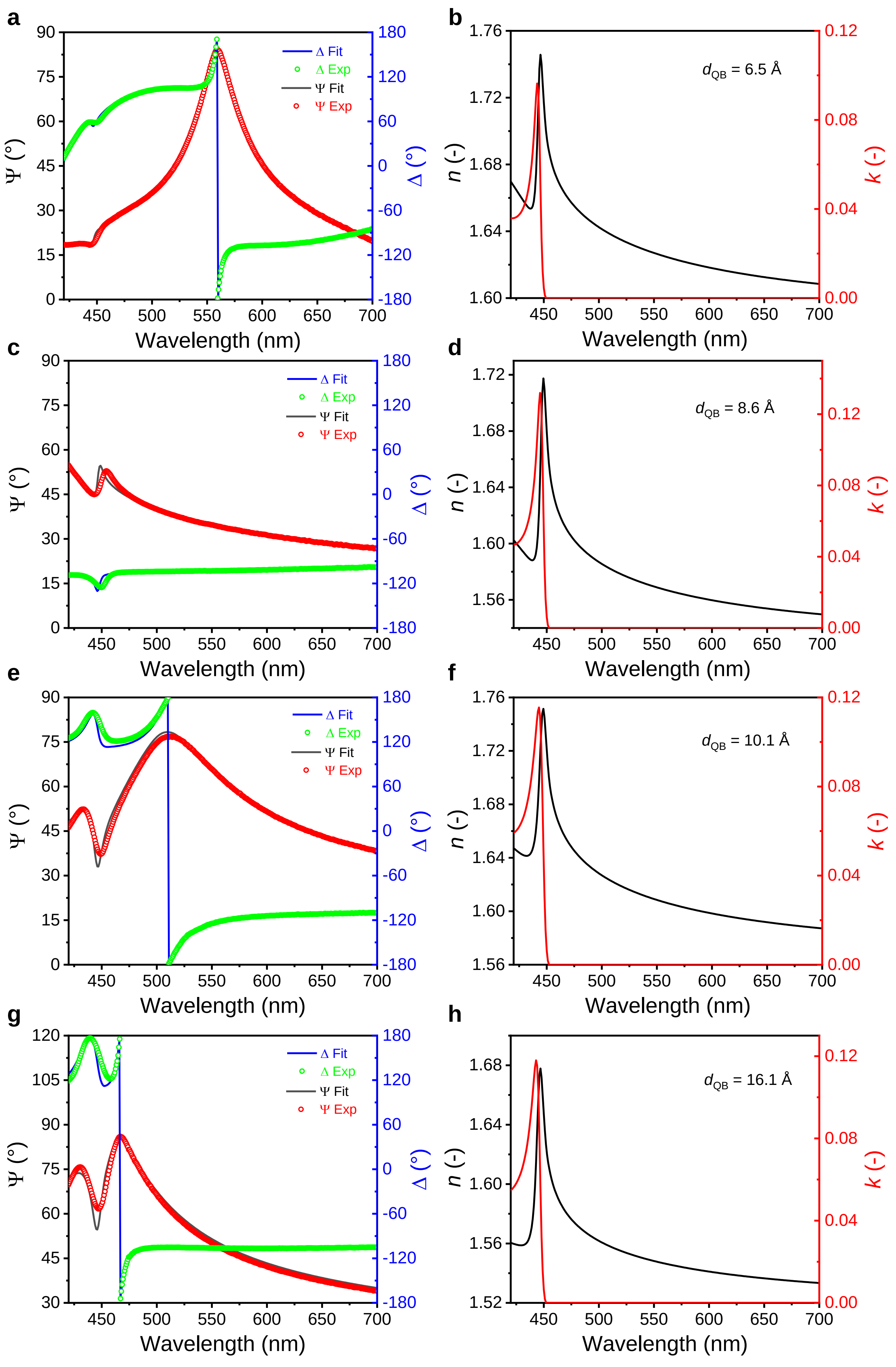
a
b
c
d
e
f
g
h
Wavelength (nm)
Ψ (°)
Δ (°)
n (-)
k (-)
Δ Fit
Δ Exp
Ψ Fit
Ψ Exp
d_QB = 6.5 Å
d_QB = 8.6 Å
d_QB = 10.1 Å
d_QB = 16.1 Å

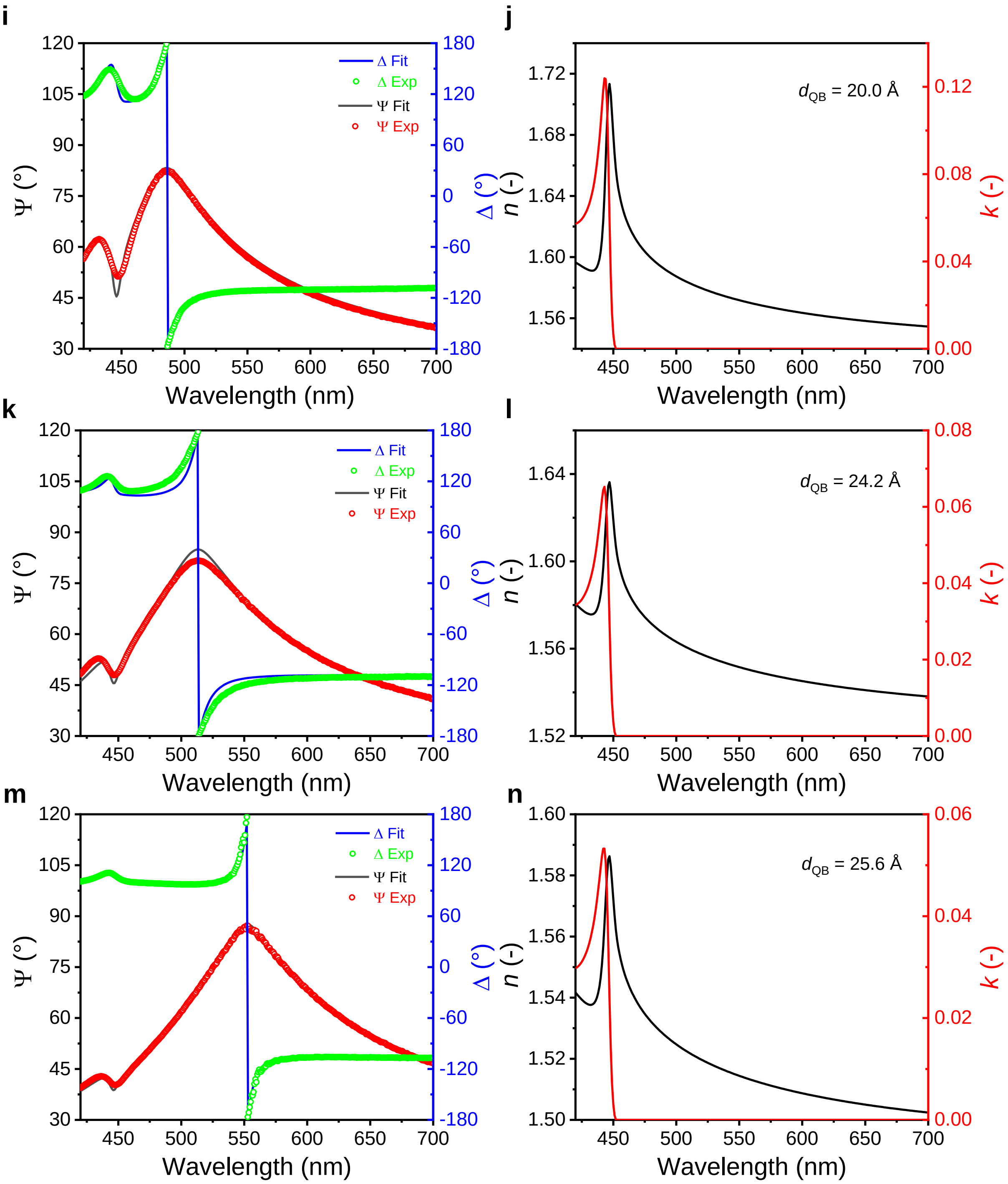

Supplementary Figure 8 | Spectroscopic ellipsometry analysis. Spectroscopic ellipsometry was employed to quantify the thickness and refractive index of superlattices made by perovskite CQWs with variable d_{QB} (**a** – **n**, increasing from top to bottom). In order to account for optical absorption of CQWs, Tauc-Lorentz model was used to fit amplitude component Ψ (black) and phase shift Δ (blue) as a function of incident light wavelength (left panels). As a result a relation between refractive index and wavelength was obtained (right panels). *n* and *k* stand for the real and imaginary component of the refractive index, respectively. Generally, a gradual decrease of the refractive index is observed, due to increased volume fraction of hydrocarbons in superlattices with larger d_{QB}. All fitting parameters were shown in Supplementary Table 1.

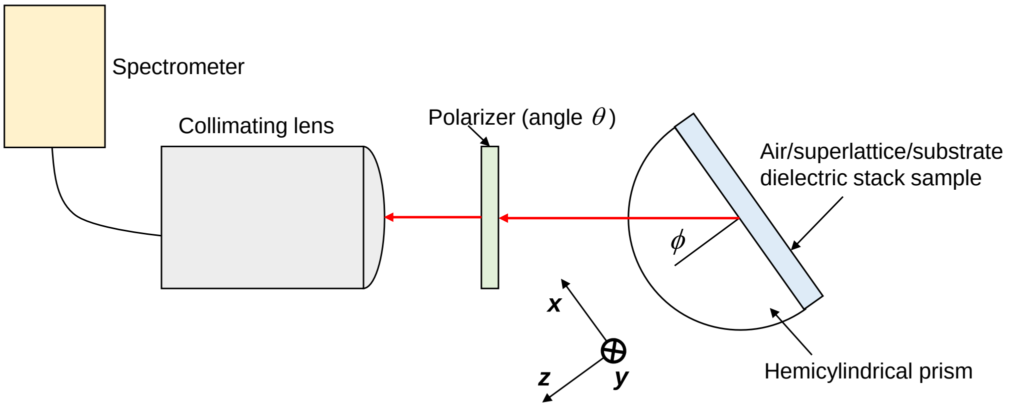

Supplementary Figure 9 | Schematic of polarization- and angle-dependent PL measurement setup using Fluxim Phelos model.

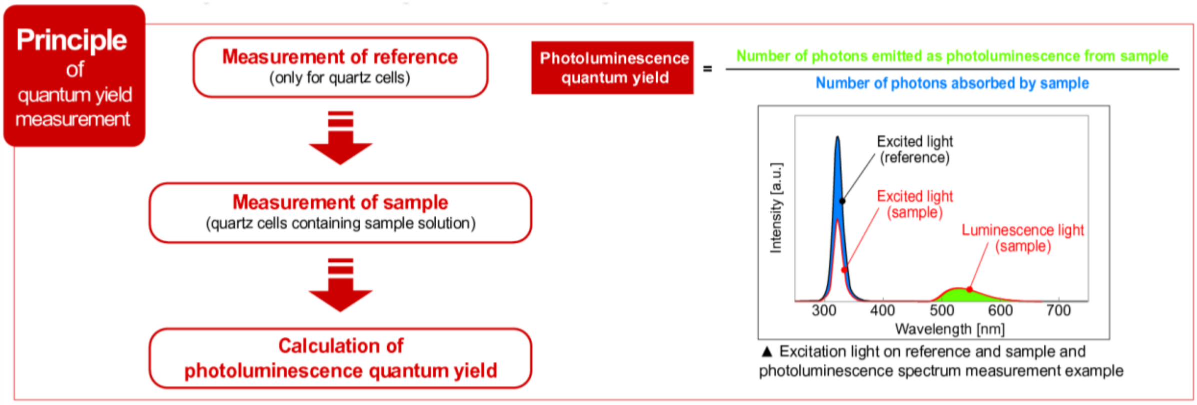

Supplementary Figure 10 | Simplified schematic presenting the principle of PLQY measurement using Hamamatsu Quantaurus QY spectrophotometer.

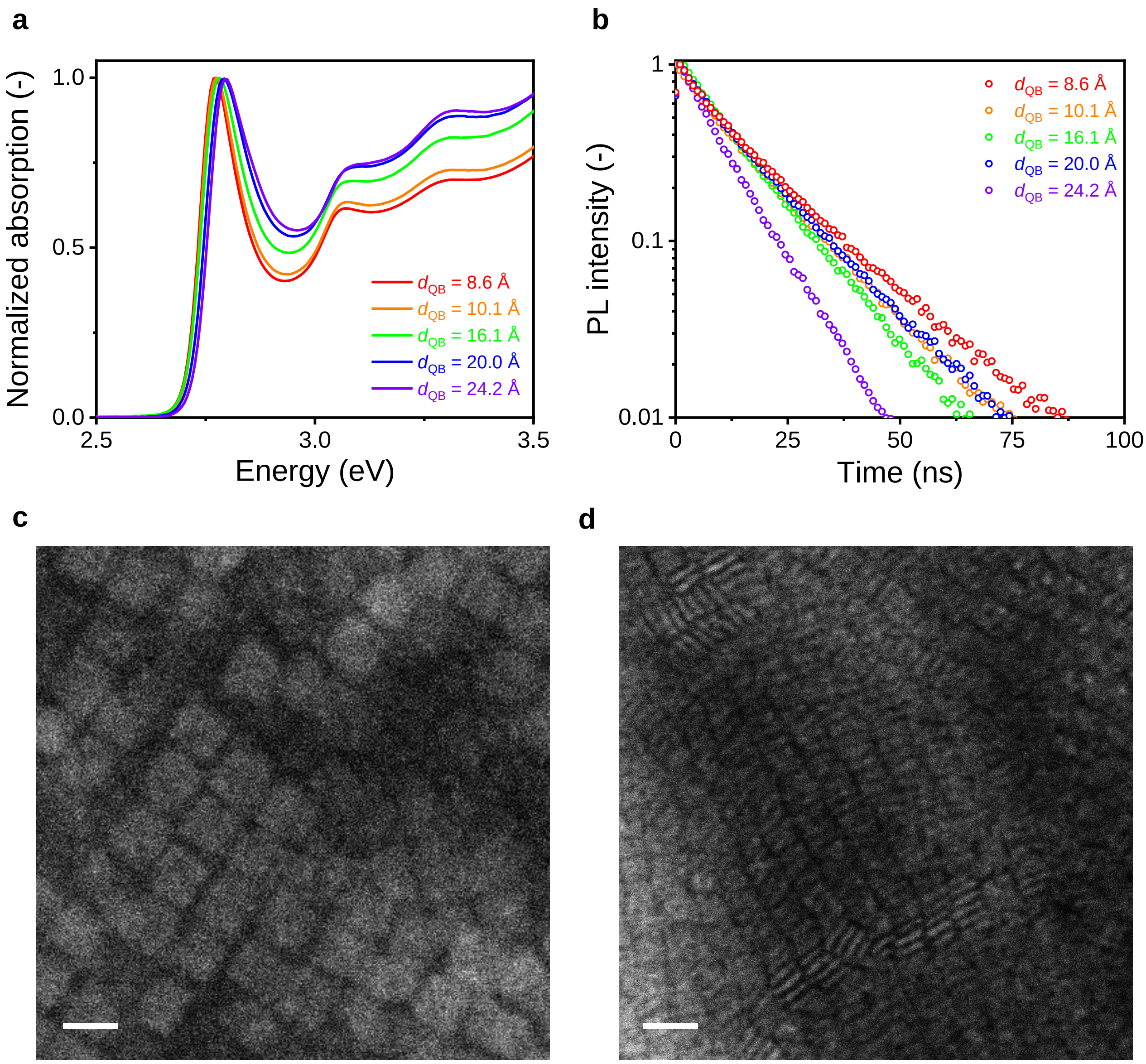

Supplementary Figure 11 | The effects of decreased CQW lateral size. a, Normalized absorption spectra acquired for colloidal dispersions of CQWs with varying d_{QB}. A gradual increase of band gap energy can be observed for larger quantum barriers. In parallel, TRPL analysis (**b**) indicates shorter lifetime for superlattice with d_{QB} = 24.2Å. Both effects are attributed to the significantly reduced lateral size of CQWs upon introducing the longest saturated alkyl group. TEM image taken for nanoparticles with small interlayer separation (d_{QB} = 10.1Å) (**c**) reveals strucutres which are ~20 nm wide, whereas the ones with large interlayer distance (d_{QB} = 24.2Å) (**d**) are approximately 2 times smaller, which is close to the Bohr diameter of this material. As a consquence, stronger exciton confinement in the in-plane directions of the QW is expected, which leads to an increased band gap energy and influences the exciton orientation within QWs. Scale bar length always corresponds to 20 nm.

Supplementary Table 1 | Absolute η_{PL} values as a function of d_{QB}.

N (-)	d_{QB} (Å)	η_{PL} (%)	Standard deviation (%)
1	8.6	48.6	0.7
1	16.1	69.1	0.7
1	25.6	79.4	0.8
2	6.5	36.7	0.9
2	8.6	52.4	0.6
2	10.1	58.3	1.8
2	16.1	62.2	0.3
2	20.0	73.1	0.8
2	24.2	79.3	1.6
2	25.6	82.2	0.6
3	6.5	40.9	1.1
4	10.1	59.1	0.8
4	20.0	76.7	1.2
4	25.6	81.5	0.9
5	24.2	75.1	0.6
6	8.6	57.1	0.3
6	16.1	60.8	0.6
7	10.1	65.2	1.1
7	20.0	75.6	0.9
8	6.5	45.6	1.3
9	24.2	74.3	0.9
10	25.6	78.0	0.3
13	16.1	59.9	1.0
14	8.6	52.8	1.8
16	10.1	60.3	2.8
17	6.5	44.6	0.2
17	20.0	71.4	0.3
18	24.2	71.6	0.9
19	25.6	75.0	0.4

Supplementary Table 2 | Dipole transition matrix elements for n = 0 calculated at G_0W_0 level for VBM-CBM transition.

M-Point	XX (IP)	YY (IP)	ZZ (OP)
	1.70318620170698	1.70318620170698	6.40312423743285E-10

Supplementary Table 3 | Dipole transition matrix elements for bulk calculated at G_0W_0 level for VBM-CBM transition at the R- and M-point. No preferential orientation is observed for different polarizations, with a slightly higher contribution at *M* for XX and YY. Such excitation is dominant in thin layers as noticed in the table above.

R-Point	XX	YY	ZZ
	1.3962871368746	1.3962871368746	1.3958904841433
M-Point	XX	YY	ZZ
	7.84564669163734E-06	7.84455121979581E-06	2.33754144348287E-08

Supplementary Table 4 | Full description and amounts of alkyl amines used for synthesis of CQWs.

Abbreviation	Full name	Amount
PAm	Pentylamine	0.173 mL
HAm	Hexylamine	0.200 mL
OAm	Octylamine	0.250 mL
DAm	Decylamine	0.300 mL
DDAm	Dodecylamine	0.350 mL (molten)
TDAm	Tetradecylamine	322.2 mg
OLAm	Oleylamine	0.500 mL

Supplementary Table 5 | Sellmeier parameters obtained from fitting the SE data for CQWs with variable d_{QB}.

d_{QB} (Å)	A_1 (-)	A_2 (-)	A_3 (-)	B_1 (μm^2)	B_2 (μm^2)	B_3 (μm^2)
6.5	0.0	1.81230	9.11088	0.04508	0.00001	8.96369
8.6	1.47596	0.0	0.00001	0.00	15.70323	150.79022
10.1	1.34167	0.0	0.0	0.04834	3.43249	753.00378
16.1	1.31099	0.0	0.00064	0.01929	0.177223	173.33187
20.0	1.42782	0.0	0.30050	0.01471	17.98699	604.22039
24.2	1.35186	0.0	0.0	0.02811	0.99617	796.98626
25.6	1.25657	0.03654	0.68737	0.01754	0.17735	100.52632

Supplementary Table 6 | Tauc-Lorentz parameters obtained from fitting the SE data for superlattices with different d_{QB}.

d_{QB} (Å)	ε_∞(-)	E_g (eV)	E_1 (eV)	TA_1 (eV)	TC_1 (eV)	E_2 (eV)	TA_2 (eV)	TC_2 (eV)
6.5	2.11	2.74	2.78	51.32	0.040	3.07	26.02	5.86
8.6	1.97	2.74	2.78	69.15	0.041	3.07	21.36	5.69
10.1	2.03	2.74	2.78	81.65	0.047	3.07	25.03	5.60
16.1	2.01	2.74	2.78	68.14	0.052	3.07	21.83	5.58
20.0	2.07	2.74	2.78	71.26	0.049	3.07	21.73	3.30
24.2	2.10	2.74	2.78	38.42	0.056	3.07	9.83	4.98
25.6	2.02	2.74	2.78	31.20	0.059	3.07	7.88	5.90

Supplementary Table 7 | Maximum η_{PL} values obtained for colloidal dispersions and superlattices of CQWs with variable d_{QB}.

d_{QB} (Å)	$\eta_{PL,film}$ (%)	$\eta_{PL,solution}$ (%)
6.5	45.6 ± 1.3	80.0 ± 0.3
8.6	67.6 ± 1.2	88.3 ± 0.1
10.1	80.2 ± 0.8	86.2 ± 0.2
16.1	78.3 ± 1.0	84.4 ± 0.1
20.0	80.3 ± 0.9	73.2 ± 0.5
24.2	79.3 ± 1.6	78.6 ± 0.1
25.6	84.7 ± 0.4	75.5 ± 0.3
